# Racial or Ethnic Health Disparities among Older Adults in Four Population Groups in South Africa

**DOI:** 10.29024/aogh.13

**Published:** 2018-04-30

**Authors:** Nancy Phaswana-Mafuya, Karl Peltzer

**Affiliations:** 1 Office of Deputy Vice Chancellor, Research and Innovation, North West University, Potchestroom, ZA; 2 HIV/AIDS/STIs and TB (HAST), Human Sciences Research Council, Pretoria 0001, ZA; 3 Department of Research and Innovation, University of Limpopo, Sovenga 0727, ZA

## Abstract

**Background:**

Racial or ethnic health disparities have been evidently apparent during the apartheid era in South Africa. This study aims to assess ethnic health disparities in four elderly population groups.

**Methods:**

Data for this study emanated from the 2008 study of “Global AGEing and adult health (SAGE) wave 1” (N = 3284) aged 50 years or older in South Africa. Associations between exposure variables and outcome variables (health status variables and chronic conditions) were examined through bivariate analyses and multivariable logistic regression.

**Results:**

Indians or Asians reported the highest prevalence of poor self-rated health (23.7%) and functional disability (11.6% and 29.1%). Coloureds had the lowest grip strength (55.3%) and Whites the highest cognitive functioning (80.1%). Coloureds had the highest prevalence of hypertension (85.0%), stroke and/or angina (15.0%), edentulism (26.8%) and low vision (50.6%); and Indians or Asians had the highest prevalence of arthritis (43.5%) and diabetes (24.4%). In adjusted analysis, Whites (Odds Ratio [OR]: 0.24, Confidence Interval [CI]: 0.11, 0.57) and Coloureds (OR: 0.50, CI: 0.29, 0.87) had lower odds of self-reported health status compared to Black Africans. Coloureds (OR: 0.36, CI: 0.22, 0.61) had lower odds of grip strength than Black Africans. Indians or Asians had higher odds of functional disability (OR: 1.87, CI: 1.03, 3.02) and diabetes (OR: 2.65, CI: 1.45, 4.83) than Black Africans. Whites (OR: 3.92, CI: 1.63, 9.41) and Coloureds (OR: 2.14, CI: 1.21, 3.78) had higher odds of cognitive functioning than Black Africans. Whites had lower odds (OR: 0.54, CI: 0.31, 0.93) and Indians or Asians had higher odds (OR: 1.91, CI: 1.91, 1.01, 3.59) of arthritis than Black Africans. Coloureds had a higher prevalence of hypertension (OR: 1.71, CI: 1.14, 2.58), stroke and/or angina (OR: 1.74, CI = 1.36, 2.22), edentulism (OR: 6.51, CI: 4.07, 10.41) and low vision (OR: 1.68, CI: 1.29, 2.19) than Black Africans.

**Conclusion:**

There are still ethnic health disparities in South Africa in the post-apartheid era (i.e., Black Africans [lower cognitive functioning], Whites [poor self-reported health status and edentulism], Coloureds [poor self-reported health status, lower grip strength, arthritis, hypertension, stroke and/or angina, edentulism and low vision], Indians or Asians [poor functional disability, arthritis and diabetes]). Understanding these ethnic health disparities may help in developing better strategies to improve health across population groups.

## Introduction

According to the population health model, health outcomes may vary by race or ethnicity, socioeconomic status, geography and gender [1]. Racial or ethnic health differences are at the centre of public health efforts in many parts of the world [2,3]. While racial or ethnic health disparities among (older) adults have been extensively studied in developed countries, particularly the United States, little is known about ethnic health disparities in relation to health status and chronic conditions in South Africa in the post-apartheid era. There were well-documented significant health disparities among racial or ethnic groups in apartheid South Africa [4]. Understanding racial or ethnic health disparities in the post-apartheid era is crucial for developing effective strategies to improve health [5].

Ethnic health disparities have been found in terms of health status. Regarding self-rated health status, older White Americans self-rated their health status higher than older Black Americans [6]; similarly, White South Africarns self-rated their health higher than Black South Africans in 1999 [7]. In a study among six ethnic groups in Amsterdam, handgrip strength (as an indicator of physical health) was higher among the Dutch nationals than among all other migrant ethnic groups, with the South-Asian Surinamese ethnic group reporting the lowest handgrip strength [8]. In a review, grip strength in developing regions was generally lower than in developed countries [9]. In terms of functional disability, disability-activities of daily living (ADLs) were reported at a higher rate among older Blacks than Whites [10]. Lin et al. [11]. found among older American adults, “the crossover ADL and instrumental activities of daily living (IADL) disparities (whites > blacks) occurring at age 75 increased with age and reached a plateau at age of 80.” Regarding cognitive functioning, older Black Americans scored lower than older White Americans on cognition level [11,12], and Lee et al. [13]. found a greater cognitive decline in mild cognitive impairment among African Americans than in non-African Americans.

Regarding chronic conditions, various studies found that African Americans were at greater risk than Caucasian Americans for hypertension [14,15] and cardiovascular disorders, including stroke [10,16] and diabetes [10,15,17]. Among studied ethnic groups in Europe, diabetes prevalence was significantly higher in South-Asian and Blacks than in Whites [18]. Blacks in North America were more likely to be obese than Whites [10,17,19]. The prevalence of arthritis was higher among Whites than among Blacks and Hispanics in the United States [20]. In a study about arthritis, the health disparities of Native Hawaiians and Pacific Islanders were higher than in Whites and Asians [21]. Asthma prevalence was the highest among Native Americans and Blacks and the lowest among Asian and Hispanic adults [22]. The highest prevalence of asthma was found among Puerto Ricans, followed by other Hispanics and Blacks, and Indians or Asians had the lowest prevalence of asthma [23]. A study in London found chronic obstructive pulmonary disease (COPD) was less likely to occur in Blacks and Asians than in Whites [24]. In an aging population, African Americans had higher rates of respiratory impairment than White Americans [25]. White Americans had a higher likelihood of being edentulous than Black Americans [26], Chinese and other Asians [27]. Whites were more likely to report vision problems than Blacks in the United States [10]. Further, major depression was less prevalent in Black Americans than White Americans [28,29], and psychological distress was higher in all “Black groups (Black African, Coloured and Indian or Asian)” than Whites in South Africa in 2002–2004 [30].

South Africa is one of the most diverse societies with a complex mix of races, cultural identities, languages and ethnic bonds. This study provides a general picture of ethnic health disparities in relation to health status and chronic conditions among Black African, White, Coloured (mixed race) and Asian people in a national probability sample of older South Africans who participated in the study on global AGEing and adult health (SAGE). Population-based surveys provide an important source of information through which ethnic variations in health status and chronic conditions can be more thoroughly understood and responsive interventions can be developed [31].

## Methods

### Sample and procedure

Data comes from the 2008 population-based cross-sectional study involving a sample of 3,840 persons aged 50 years or older in South Africa. The SAGE sample design entails a two-stage probability sample that yields national and subnational estimates to an acceptable precision at provincial level, by locality type (urban and rural), and by population group (including black, coloured [mixed ancestry], Indian or Asian and white) [32]. The individual response rate among those aged 50 years or older was 77%. The study on global AGEing (SAGE) survey was carried out in South Africa in partnership between the World Health Organization (WHO), the National Department of Health, and the Human Sciences Research Council (HSRC) [32]. The study was approved by the Human Sciences Research Council Research Ethics Committee (Protocol REC 5/13/04/06) and the national Department of Health, and written informed consent was obtained from participants.

### Measures

*Racial groups*. Participants were asked, “What is your background or racial group?” The response options were “Black African, Coloured, Indian/Asian, White and other;” those that responded “other” were not included in this analysis. According to Statistics South Africa [33] “in South Africa, analysis by the four population groups remains an important crosscutting variable to better understand and plan for education, employment, health, mortality, fertility, and migration within the country.”

*Age, gender, educational level, geolocation and economic or wealth status* of a given household was estimated based on a list of household assets, and subsequently, wealth quintiles were created from these [34].

*Health risk behaviours* assessed included daily tobacco use [35], problem drinking [36], physical inactivity [37] and inadequate fruit and vegetable consumption (<5 servings a day) [38].

*Health status variables* included self-reported health status [32], grip strength [39], functional disability using the 12-item “WHO Disability Assessment Schedule, version 2 (WHODAS-II)” [40,41] and cognitive functioning based on a battery of cognitive tests [42].

*Chronic conditions* were assessed as follows: arthritis (symptoms algorithm based) [43], asthma (self-reported diagnosed and/or symptoms algorithm based) [43], lung disease (symptoms algorithm based) [43], obesity (standard height and weight measures) [32] and diabetes, stroke, angina and edentulism (self-reported diagnosed) [32].

*Blood pressure (systolic and diastolic)* was assessed “three times on the right arm/wrist of the seated respondent using an automated recording device (OMRON R6 Wrist Blood Pressure Monitor, HEM-6000-E, Omron Healthcare Europe, B.V., Hoofddorp, and The Netherlands) [32].” Of the three blood pressure readings, the average of the last two readings was used [32]. Participants with “systolic blood pressure ≥ 140 mmHg and/or diastolic blood pressure ≥ 90 mmHg and/or who reported the current use of antihypertensive medication” were classified as having high blood pressure [44].

*Depression*. The World Mental Health Survey version of the “Composite International Diagnostic Interview” was used to assess symptom-based depression in the past 12 months [45]. The depression diagnosis was based on the “International Classification of Diseases” (ICD-10) [46], using an algorithm taking into account depressive symptoms in the past 12 months [47]. In addition, respondents who agreed to the question, “Have you been taking any medications or other treatment such as attending therapy or counselling sessions for depression during the last 12 months?” were added to the symptom-based diagnosis of depression.

*Vision* was measured using a tumbling “E” log MAR chart; visual acuity was assessed for both near and far vision in both eyes [48]. Assessed near and distance visual acuity was categorized into “low vision (0.01–0.25 decimal) and normal vision (0.32–1.6 decimal)” [49]. In this investigation, a study participant had low vision if he or she had either low near or far vision in both eyes.

### Data analysis

Using STATA software version 13.0 (Stata Corporation, College Station, Texas, USA) data were analysed taking into account the sampling design. Chi-square tests were used to test differences in proportions of exposure variables across different populations groups. Associations between exposure variables (sociodemographics and health risk behaviours) and outcome variables (health status variables and chronic conditions) were examined through calculation of odds ratios (OR) using logistic regression. In the tables, weighted percentages are presented. The p-value of less than 5% indicated statistical significance. The p-values and 95% confidence intervals were adjusted for the complex sample design of the study.

## Results

### Sample characteristics

The sample included in this study was 3,284 individuals 50 years or older (44.1% men and 55.9% women). The most populous racial or ethnic group was Black African (74.0%), followed by Coloureds (12.8%), Whites (9.3%) and Indians or Asians (3.8%). Compared to other racial or ethnic groups, Black Africans had the lowest economic or wealth status, had the lowest formal education, and were more likely to live in rural areas. Coloureds seemed to have a higher prevalence of health risk behaviours, such as daily tobacco use, insufficient fruit and vegetable consumption and physical inactivity, than the other population groups (see Table 1).

**Table 1:** Exposure variables by population group (N = 3284).

Variable	Black African n = 1982	White African n = 253	Multi-Ancestry n = 637	Indian/Asian African n = 287	p-Value

	M (SD)	M (SD)	M (SD)	M (SD)	

Age	61.3 (9.5)	62.8 (9.0)	61.7 (9.6)	62.3 (8.1)	0.261

	n (%)	n (%)	n (%)	n (%)	

Gender					
Female	1250 (60.0)	137 (53.6)	423 (63.2)	171 (61.1)	0.522
Male	803 (40.0)	132 (46.4)	232 (36.8)	136 (38.9)	
Education					
< 7 years	1241 (60.5)	12 (3.7)	318 (42.1)	111 (34.5)	< 0.001
8–11	536 (29.2)	112 (38.2)	265 (44.3)	134 (37.3)	
12 or more	195 (10.3)	131 (58.1)	34 (13.7)	51 (28.2)	
Wealth					
Low	1055 (50.6)	8 (3.0)	212 (24.8)	23 (13.1)	< 0.001
Medium	416 (21.0)	14 (4.3)	165 (18.3)	53 (14.9)	
High	570 (28.4)	245 (92.7)	277 (56.9)	230 (72.1)	
Residence					
Urban	1131 (56.5)	218 (84.9)	545 (89.6)	285 (86.2)	0.003
Rural	920 (43.5)	51 (15.1)	110 (10.4)	22 (13.8)	
Marital status					
Not married, single	1059 (49.7)	82 (31.5)	333 (46.2)	115 (45.5)	0.018
Married, cohabiting	952 (50.3)	181 (68.5)	313 (53.8)	191 (54.5)	
Daily tobacco use	378 (17.7)	58 (21.8)	205 (34.1)	56 (19.3)	0.006
Problem drinking	91 (4.0)	7 (4.5)	33 (4.5)	9 (1.8)	0.706
Physical inactivity	1207 (57.7)	169 (55.7)	518 (76.9)	182 (52.3)	0.005
Inadequate fruit and vegetable consumption	1580 (72.3)	158 (47.7)	511 (73.7)	185 (54.6)	0.007

### Health status and chronic conditions

Compared to other population groups, Indians or Asians reported the highest prevalence of poor self-rated health (23.7%), severe ADL (11.6%) and severe IADL (29.1%). Coloureds had the lowest handgrip strength (55.3%) among the other population groups. Cognitive function was highest among Whites (80.1%) compared to the other groups.

Compared to other population groups, Coloureds had the highest prevalence of hypertension (85.0%), stroke and/or angina (15.0%), edentulism (26.8%) and low vision (50.6%). Indians or Asians had the highest prevalence of arthritis (43.5%) and diabetes (24.4%). Whites were found to have the lowest prevalence of asthma (8.4%), and Indians or Asians had the lowest prevalence of obesity (38.7%). A similar prevalence of lung disease and depression was found across racial groups (see Table 2).

**Table 2:** Prevalence of health status and chronic conditions by population group.

Variable	Black African n (%)	White African n (%)	Multi-Ancestry n (%)	Indian/Asian African n (%)	p-Value

**Health status**					

Poor self-rated health	362 (19.6)	21 (4.0)	99 (10.0)	57 (23.7)	< 0.001
Grip strength	1472 (78.5)	204 (81.1)	417 (55.3)	178 (71.4)	< 0.001
ADL-severe	85 (4.7)	12 (4.4)	29 (5.0)	33 (11.6)	0.409
IADL-severe	346 (19.7)	27 (7.8)	97 (20.1)	67 (29.1)	0.007
Cognitive functioning	798 (44.5)	211 (80.1)	342 (60.5)	176 (64.7)	< 0.001
**Chronic conditions**					

Arthritis	536 (27.3)	52 (18.7)	214 (39.1)	111 (43.5)	< 0.001
Asthma	192 (11.8)	15 (8.4)	62 (11.2)	46 (12.8)	0.654
Lung disease	124 (6.0)	15 (7.1)	37 (6.4)	29 (6.2)	0.961
Hypertension	1550 (77.3)	193 (79.6)	545 (85.0)	214 (76.8)	0.147
Obesity	843 (45.3)	110 (46.7)	245 (46.8)	104 (38.7)	0.814
Diabetes	149 (7.8)	23 (12.0)	68 (10.5)	69 (24.4)	0.021
Stroke and/or angina	138 (7.1)	28 (9.0)	73 (15.0)	48 (11.5)	< 0.001
Depression	57 (3.2)	9 (2.1)	21 (1.3)	14 (3.3)	0.319
Edentulism	90 (4.1)	54 (18.0)	145 (26.8)	33 (5.1)	< 0.001
Low vision	769 (41.2)	92 (39.2)	323 (50.6)	123 (41.5)	0.148

### Odds ratios for health status and chronic conditions by population group

In adjusted analysis (with sociodemographic factors and health risk behaviour variables), compared to Black Africans, Whites and Coloureds had a lower self-reported health status. Further, Coloureds had a lower grip strength than Black Africans. Indians or Asians were more likely to have severe ADL and IADL, and Whites were less likely to have severe IADL than Black Africans. Cognitive functioning was significantly higher among Whites and Coloureds than Black Africans.

Coloureds and Indians or Asians had higher odds of arthritis than Black Africans. The prevalence of asthma, lung disease and depression did not differ by population group. Coloureds had a higher prevalence of hypertension, stroke and/or angina and low vision than Black Africans. Indians or Asians were less likely to be obese than Black Africans. Edentulism was significantly higher among Coloureds and Whites than Black Africans (see Table 3).

**Table 3:** Odds ratios for health status and chronic conditions by population group.

	Black African (n = 1982)	White (n = 253)		Coloured (n = 637)		Indian or Asian (n = 287)	

		OR (95% CI)	P value	OR (95% CI)	P value	OR (95% CI)	P value

**Health status**							

*Poor self-rated health*							
Unadjusted	1 (Reference)	0.17 (0.07, 0.39)	<0.001	0.46 (0.23, 0.89)	0.023	1.27 (0.76, 2.12)	0.346
Adjusted^1^	1 (Reference)	0.24 (0.11, 0.57)	0.002	0.50 (0.29, 0.87)	0.016	1.70 (0.89, 3.24)	0.1.08
*Grip strength*							
Unadjusted	1 (Reference)	1.18 (0.57, 2.41)	0.642	0.34 (0.21, 0.56)	<0.001	0.68 (0.43, 1.10)	0.112
Adjusted^1^	1 (Reference)	1.02 (0.47, 2.22)	0.950	0.36 (0.22, 0.61)	<0.001	0.68 (0.42, 1.11)	0.118
*ADL-severe*							
Unadjusted	1 (Reference)	0.95 (0.34, 2.61)	0.915	1.08 (0.25, 4.60)	0.914	2.68 (0.94, 7.68)	0.065
Adjusted^1^	1 (Reference)	1.04 (0.42, 2.58)	0.927	1.08 (0.34, 3.46)	0.889	3.27 (1.09, 9.80)	0.035
*IADL-severe*							
Unadjusted	1 (Reference)	0.34 (0.17, 0.69)	0.003	1.02 (0.61, 1.71)	0.925	1.67 (1.07, 2.59)	0.025
Adjusted^1^	1 (Reference)	0.32 (0.14, 0.72)	0.007	0.97 (0.58, 1.61)	0.902	1.87 (1.09, 3.22)	0.025
*Cognitive functioning*							
Unadjusted	1 (Reference)	5.04 (2.13, 11.91)	<0.001	1.92 (1.18, 3.11)	0.010	2.28 (1.28, 4.05)	0.006
Adjusted^1^	1 (Reference)	3.92 (1.63, 9.41)	0.003	2.14 (1.21, 3.78)	0.010	1.81 (0.86, 3.80)	0.113
**Chronic conditions**							

*Arthritis*							
Unadjusted	1 (Reference)	0.61 (0.37, 1.00)	0.050	1.71 (1.20, 2.42)	0.004	2.05 (1.16.3.61)	0.015
Adjusted^1^	1 (Reference)	0.54 (0.31, 0.93)	0.027	1.42 (0.98, 2.04)	0.061	1.91 (1.01, 3.59)	0.046
*Asthma*							
Unadjusted	1 (Reference)	0.68 (0.27, 1.71)	0.410	0.94 (0.66, 1.35)	0.751	1.09 (0.51, 2.36)	0.814
Adjusted^1^	1 (Reference)	0.60 (0.22, 1.61)	0.302	0.87 (0.61, 1.24)	0.436	0.99 (0.43, 2.26)	0.978
*Lung disease*							
Unadjusted	1 (Reference)	1.20 (0.51, 2.79)	0.669	1.07 (0.55, 2.08)	0.848	1.04 (0.34, 3.15)	0.949
Adjusted^1^	1 (Reference)	1.10 (0.47, 2.59)	0.823	0.99 (0.50, 2.00)	0.987	0.92 (0.27, 3.22)	0.900
*Hypertension*							
Unadjusted	1 (Reference)	1.44 (0.64, 2.05)	0.640	1.66 (1.08, 2.56)	0.022	0.97 (0.59, 1.60)	0.913
Adjusted^1^	1 (Reference)	0.99 (0.51, 1.92)	0.964	1.71 (1.14, 2.55)	0.010	0.84 (0.56, 1.25)	0.376
*Obesity*							
Unadjusted	1 (Reference)	1.06 (0.64, 1.75)	0.824	1.10 (0.69, 1.77)	0.682	0.75 (0.48, 1.18)	0.209
Adjusted^1^	1 (Reference)	0.72 (0.43, 1.22)	0.220	0.80 (0.52, 1.22)	0.282	0.55 (0.33, 0.92)	0.024
*Diabetes*							
Unadjusted	1 (Reference)	1.60 (0.64, 3.99)	0.302	1.37 (0.74, 2.56)	0.307	3.80 (1.96, 7.36)	<0.001
Adjusted^1^	1 (Reference)	1.12 (0.50, 2.53)	0.783	0.99 (0.62, 1.57)	0.964	2.65 (1.45, 4.83)	0.002
*Stroke and/or angina*							
Unadjusted	1 (Reference)	1.30 (0.67, 2.51)	0.430	2.32 (1.73, 3.12)	<0.001	1.71 (0.93, 3.13)	0.083
Adjusted^1^	1 (Reference)	0.91 (0.47, 1.76)	0.771	1.74 (1.36, 2.22)	<0.001	1.36 (0.69, 2.71)	0.367
*Depression*							
Unadjusted	1 (Reference)	0.72 (0.30, 1.72)	0.458	0.63 (0.34, 1.17)	0.140	1.02 (0.36, 2.87)	0.976
Adjusted^1^	1 (Reference)	0.73 (0.23, 2.29)	0.579	0.49 (0.23, 1.02)	0.056	1.04 (0.33, 3.23)	0.947
*Edentulism*							
Unadjusted	1 (Reference)	5.16 (2.49, 10.07)	<0.001	8.56 (5.01, 14.70)	<0.001	1.27 (0.63, 2.55)	0.492
Adjusted^1^	1 (Reference)	4.13 (1.73, 9.87)	0.002	6.51 (4.07, 10.41)	<0.001	1.03 (0.50, 2.09)	0.944
*Low vision*							
Unadjusted	1 (Reference)	0.92 (0.53, 1.61)	0.768	1.46 (1.06, 2.02)	0.021	1.01 (0.81, 1.27)	0.901
Adjusted^1^	1 (Reference)	1.05 (0.56, 1.96)	0.873	1.68 (1.29, 2.19)	<0.001	1.11 (0.82, 1.51)	0.482

OR = Odds Ratio, CI = Confidence Interval; ^1^Adjusted for age, gender, education, wealth, residence, tobacco use, alcohol use, physical inactivity and fruit and vegetable consumption.

## Discussion

This study found variations in health status variables and chronic conditions among elderly South Africans across different racial groups (i.e. Black African, White, Coloured and Indian or Asian) in a nationally representative sample of South Africans 50 years and older. The study found racial differences in perceived health status. Black Africans had lower cognitive functioning, Whites had poor self-reported health status and Coloureds had poor self-reported health status and lower grip strength, while Indians or Asians had poor functional disability. Whites and Coloureds viewed themselves as having poorer health status compared to Black Africans. This is contrary to findings in previous studies conducted in South Africa and the United States, where Whites exhibited higher self-rated health than Blacks [6,7]. It may be that older Whites are more pessimistic about their health status than Black Africans (health pessimism hypothesis) [6]. This may also be partially due to the lower levels of health literacy among Black Africans. Poor self-reported health status has been strongly associated with subsequent mortality risk in the US population [31,50]. Similar to the US studies, Black Africans in this study scored lower than Whites on the level of cognition [11,12,13].

Regarding chronic conditions, racial or ethnic health disparities were found for Whites (edentulism), Coloureds (arthritis, hypertension, stroke and/or angina, edentulism and low vision), and Indians or Asians (arthritis and diabetes). These findings seem to confirm, as previously reviewed by Williams et al. [51], that observed racial disparities in health status and chronic conditions mostly remained even after taking socioeconomic status into account. The findings of previous studies[10,14,15,16,17,19] that African Americans were at greater risk than Caucasian Americans for hypertension, cardiovascular disorders, including stroke, diabetes and obesity was not confirmed in this study. Like previous studies [18], this study found that Indians or Asians had a higher prevalence of diabetes than other population groups in South Africa. However, arthritis prevalence was also higher among Indians or Asians than other population groups, which seems not to conform to previous studies [21]. While other studies found racial or ethnic disparities in the prevalence of asthma [22,23], lung diseases [24,25], and depression [23,24,30], this study did not find such differences. The finding that White South Africans had a higher likelihood of being edentulous than Black South Africans is an agreement with a previous study [26].

This study had several limitations. Health variables were self-reported. The study design was cross-sectional, so no causality can be claimed.

In conclusion, it is clear that variations exist in health status and chronic conditions. Racial or ethnic health disparities were found for Black Africans (lower cognitive functioning), Whites (poor self-reported health status and edentulism), Coloureds (poor self-reported health status, lower grip strength, arthritis, hypertension, stroke and/or angina, edentulism and low vision), and Indians or Asians (poor functional disability, arthritis and diabetes). Understanding these racial health disparities may help in developing better strategies to improve health in these population groups.
